# The Functional Role of S280 within the ZYG-1 Phosphorylation Cluster During Centrosome Assembly in *C. elegans* Embryos

**DOI:** 10.17912/micropub.biology.002127

**Published:** 2026-05-01

**Authors:** Amber Christian, Sariah Richardson, Layanne Obeid, Annabel Shaffou, Joseph DiPanni, Mi Hye Song

**Affiliations:** 1 Biological Sciences, Oakland University, Rochester, MI, US

## Abstract

Proper centrosome duplication requires precise regulation of ZYG-1 . CK2-mediated phosphorylation of ZYG-1 contributes to this process by controlling ZYG-1 stability and centrosome number. Previous work identified a cluster of serine residues in ZYG-1 that collectively modulate ZYG-1 activity, but the contribution of individual sites remains unclear, except for S279. Here, we examine the role of S280 using CRISPR/Cas9-generated phospho-deficient mutations in the *zyg-1 ( it25 )* background. While S279A strongly rescues the *zyg-1* phenotype, S280A alone shows no effect. However, combining S280A with S279A diminishes the rescue effect of S279A. These results suggest that S279 and S280 function cooperatively within a regulatory module, with S279 acting as an inhibitory site and S280 supporting optimal ZYG-1 activity.

**Figure 1. The ZYG-1:S280A mutation alone has minimal effect in zyg-1(it25) mutants but diminishes the rescue effect of S279A:** ( **A** ) Genetic analysis of&nbsp;the ZYG-1 phospho-mutations in the *zyg-1 ( it25 )* mutant backgrounds at 22°C and 23.5°C. Data are presented as mean ± s.d. n represents the number of progeny scored. &nbsp;( **B** ) % Embryonic lethality at 22°C (left) and 23.5°C (right) (see **A** ). Each dot indicates the percentage of dead embryos per hermaphrodite (N values in A). In the plots, the box ranges from the first to the third quartile of the data. The thick bar indicates the median, and whiskers extend 1.5 times the interquartile range. ^ns^ *p* >0.05, *** *p* <0.001 (two-tailed t-tests). ( **C** ) Quantification of monopolar (grey) and bipolar (white) spindles during the second mitosis in *zyg-1 ( it25 )* mutant backgrounds grown at 22°C, with individual phospho-mutations. Average values are presented. n is the total number of blastomeres scored. The bottom panel shows representative embryos stained for centrosomes ( ZYG-1 ), microtubules, and DNA (DAPI), illustrating monopolar (arrowheads) and bipolar (arrows) spindles at the second mitosis in the *zyg-1 ( it25 )* mutant background. ZYG-1 localization at centrosomes is cell cycle–dependent, peaking at anaphase and lowest at metaphase (Song et al., 2008). Here *,* ZYG-1 foci appear only in subsets of centrosomes due to cell cycle stage differences, not staining variability. Also note that in *zyg-1 ( it25 )* mutants, centrosomal ZYG-1 is reduced to ~40% of wild type (Medley et al., 2021), explaining the observed ZYG-1 signal. Bar, 10 μm.

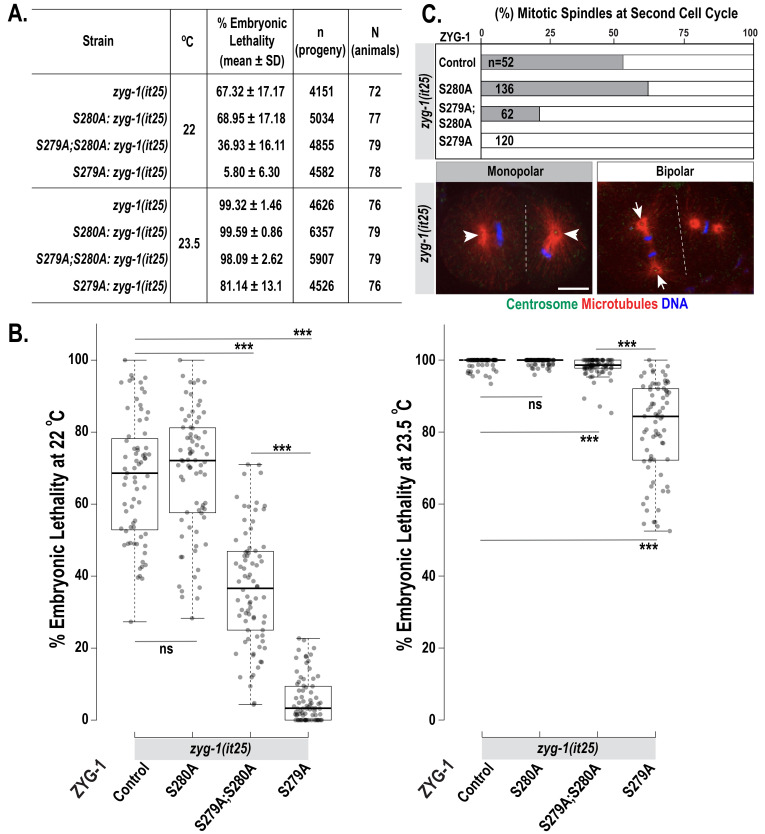



## Description

Precise regulation of centrosome assembly is essential for proper cell division. The kinase ZYG-1 /Plk4 plays a central role in this process. In *C. elegans* , CK2 negatively regulates centrosome duplication by controlling ZYG-1 levels through direct phosphorylation (Medley et al., 2017; Medley et al., 2023). Phospho-deficient ZYG-1 increases ZYG-1 levels and promotes centrosome amplification, while proteasome inhibition stabilizes phospho-mimetic ZYG-1 , indicating that CK2-dependent phosphorylation regulates ZYG-1 stability via proteolysis, thereby controlling centrosome number.

This study explores how phosphorylation at specific sites impacts ZYG-1 activity during centrosome assembly, focusing on a potential CK2 target identified in a previous study (Medley et al., 2023). Our earlier work identified a cluster of serine residues (S273, S279, S280, S285, known as ZYG-1:4S) that collectively modulate ZYG-1 activity. We found that the S279A mutation elevates centrosomal ZYG-1 levels, restoring centrosome duplication and embryonic viability *in zyg-1 ( it25 )* mutants. However, the effect of S279A was weaker than the combination of all four serine substitutions, called ZYG-1:4A (Medley et al., 2023). While mutations of the entire cluster affect centrosome assembly, the specific contributions of individual sites remain unknown, except for S279. Here, we focus on S280 within ZYG-1:4S to understand how substituting S280 with alanine affects ZYG-1 activity during centrosome duplication in early *C. elegans* embryos.

To examine the functional impact of S280, we introduced phospho-deficient mutations (S280A and S279;S280) in *zyg-1 ( it25 )* background at the endogenous locus by CRISPR/Cas9 editing. We first examined how the S280A mutation might influence embryonic lethality in the hypomorphic *zyg-1 ( it25 )* mutant background. The *zyg-1 ( it25 )* allele is a recessive, temperature-sensitive mutation that causes a highly penetrant embryonic-lethal phenotype (100%) at 24°C (O'Connell et al., 2001). Previous work has shown that the suppression of *zyg-1 ( it25 )* phenotypes conferred by the S279A mutation is temperature-dependent, with stronger effects at lower temperatures (Medley et al., 2023). This suggests that the S279A mutation relies on residual ZYG-1 activity to rescue the *zyg-1 &nbsp;* phenotype.&nbsp; Thus, we assessed their impact on embryonic lethality at semi-restrictive temperatures of 22°C and 23.5°C, where the hypomorphic ZYG-1 function in *zyg-1 ( it25 )* mutants remains partially active ( Figure 1A, 1B). Consistent with previous findings (Medley et al., 2023), the S279A mutation significantly reduced embryonic lethality in *zyg-1 ( it25 )* mutants (5.8 ± 6.3%, *p* <0.0001), compared to *zyg-1 ( it25 )* controls (67.32 ± 17.17%) at 22°C. In contrast, the S280A mutation showed no significant effect on embryonic lethality in *zyg-1 ( it25 )* mutants (68.95 ± 17.18%, *p* =0.564), comparable with the control *zyg-1 ( it25 )* mutants (67.32 ± 17.17%). At 23.5°C, similar genetic relationships were observed, but with less pronounced effects: The S279A mutation decreased embryonic lethality in *zyg-1 ( it25 )* mutants (81.14 ± 13.1%, p<0.001), whereas the S280A mutation did not cause a significant change (99.59 ± 0.86%, *p* =0.161), compared to *zyg-1 ( it25 )* controls (99.32 ± 1.46%). Interestingly, the S279A; S280A double mutation produced an intermediate effect between the S279A and S280A single mutations on embryonic lethality, with lethality of 36.93 ± 16.11% at 22°C and 98.09 ± 2.62% at 23.5°C. These rates were higher than those seen with the S279A single mutation but lower than with the S280A mutation alone, indicating that S280A significantly reduces the rescue effect of S279A ( *p* <0.0001).

Since ZYG-1 is essential for centrosome duplication, rescuing embryonic lethality in *zyg-1 ( it25 )* mutants likely occurs through successful centrosome duplication. To examine how the S280A mutation influences this process, we stained embryos with antibodies for the centrosome marker ZYG-1 and microtubules, then scored mitotic spindle formation during the second mitotic division ( Figure 1C ). Because the rescue effect of S279A on embryonic lethality was more potent at 22°C than at 23.5°C, we used the 22°C condition to examine centrosome duplication and detect any subtle rescue effects on spindle formation. At this temperature, control *zyg-1 ( it25 )* embryos showed 52% monopolar spindles (n=52), indicating residual ZYG-1 activity under these conditions. Consistent with the genetic analysis (Figures 1A, 1B), the S280A mutation did not rescue but slightly increased monopolar spindles to 61% (n=136), while the S279A mutation rescued monopolar spindles (0%, n=120). The double mutant S279A; S280A resulted in 79% bipolar spindles (n=62), an intermediate between the single mutations.&nbsp;

These results show that the S280A alone does not rescue the centrosome duplication defect or embryonic lethality in *zyg-1 ( it25 )* mutants, whereas S279A strongly rescues the mutant phenotype. However, combining S280A with S279A significantly reduces the rescue effect of S279A, indicating that S280 is required for maximal rescue by S279A. These findings suggest that S279 functions as an inhibitory regulatory site, while S280 supports the active state of ZYG-1 .&nbsp;

Together, our results indicate that S279 and S280 act as a coupled regulatory unit in ZYG-1 . Blocking phosphorylation at S279 enhances ZYG-1 activity, but this requires an intact S280, suggesting cooperative function. Consistently, CK2 phosphorylates at least one of these sites with similar efficiency (Medley et al., 2023), supporting the notion of a local regulatory module. S279A increases ZYG-1 activity by blocking inhibitory phosphorylation, whereas S280A may counteract this effect by disrupting CK2 recognition or structural context. The more potent suppression by the ZYG-1:4A mutant further highlights that multiple phosphorylation sites collaboratively regulate ZYG-1 , with combined mutations producing a coordinated change in regulatory inputs rather than a simple additive effect, collectively modulating ZYG-1 function during centrosome duplication.

## Methods

***C. elegans* Culture and Genetic Analysis:** All strains were derived from the OC14 strain and maintained on MYOB plates seeded with *Escherichia coli* OP50 at 20°C. To assess embryonic lethality, L4 animals were singled onto individual plates and allowed to self-fertilize for 24 hours at the indicated temperature. Progeny were given 24 hours to complete embryogenesis, after which the number of hatched larvae and unhatched (dead) eggs was recorded. To evaluate centrosome duplication events, L4 animals were grown for 16-18 hours at 22°C, and adult gravid worms were processed for immunostaining.

**Immunostaining and Confocal Microscopy:** Immunofluorescence and confocal microscopy were performed as described (Medley et al., 2017). For immunostaining, the following primary and secondary antibodies were used at 1:3000 dilutions: α- ZYG-1 (Stubenvoll et al., 2016), DM1a (Millipore Sigma, T9026), and Alexa Fluor 488 and 568 secondary antibodies (Thermo Fisher Scientific, A48482TR, A11004). Confocal microscopy was performed using a Nikon Eclipse Ti-U microscope equipped with a Plan Apo 60×1.4 NA lens, a Spinning Disk Confocal (CSU X1), and a Photometrics Evolve 512 camera. MetaMorph software (Molecular Devices, Sunnyvale, CA, USA) was used for image acquisition, and Adobe Photoshop/Illustrator for image processing. Co-stained embryos at the second mitosis were examined for spindle formation.

**CRISPR/Cas9 Genome Editing:** For genome editing, we used the co-CRISPR technique described (Arribere et al., 2014, Paix et al., 2015). To design crRNA, we used the CRISPOR webserver (crispor.tefor.net; Concordet and Haeussler, 2018). Animals were microinjected with a mixture of commercially available SpCas9 (IDT, Coralville, IA) and custom-designed oligonucleotides (IDT, Coralville, IA), including crRNAs at 0.4–0.8 µg/ml ( *zyg-1 :* 5'- UGGACGACGACAGAGAUCGAGUUUUAGAGCUAUGCU-3'), tracrRNA at 12 µg/ml, and single-stranded DNA oligonucleotides at 25–100 ng/ml. After injection, we screened the F1 progeny for *dpy-10 ( cn64 ) II/+* rollers and genotyped the F2 for the targeted mutation. The genome editing was verified by Sanger Sequencing (GeneWiz, South Plainfield, NJ). All the *C. elegans* strains generated in this study produce nearly 100% viable progeny at 20°C.

Single-stranded DNA oligonucleotides, homologous repair templates (IDT, Coralville, IA) for genome editing, were as follows.

**ZYG-1 :S280A** ;

5'-GAGAACACTCGCGGGATGGACGACGACAGAGATCGCGAGAACCAGTAAGATCC **GCT** AGAGATGATCGATCTCGAGATGGCAGAGCTCTGAT-3'

**ZYG-1 :S279A; S280A** ;

5'-GAGAACACTCGCGGGATGGACGACGACAGAGATCGCGAGAACCAGTAAGA **GCCGCT** AGAGATGATCGATCTCGAGATGGCAGAGCTCTGAT-3'

**Statistics:** Statistical analyses were performed using R, and data are presented as mean ± SD. Dot plots were generated using the R beeswarm package; boxes indicate the interquartile range, the median is shown as a thick line, and whiskers extend to 1.5 times the interquartile range or to the data range. Box plots were generated using BoxPlotR (Spitzer et al., 2014; http://shiny.chemgrid.org/boxplotr/ ). *p* -values were calculated using two-tailed *t* -tests: ns *p* > 0.05; *** *p* ***<* 0.001** .

## Reagents

**Table:** 

Strain	Genotype	Available
OC14	*zyg-1 ( it25 [P442L]) *	O'Connell et al., 2001
MTU25	*zyg-1 ( mhs399 it25 [S279A, P442L])II *	Medley et al., 2023
MTU842	*zyg-1 ( mhs788 it25 [S279A, S280A, P442L])II *	This study
MTU854	*zyg-1 ( mhs794 it25 [S280A, P442L])II *	This study
